# Epidemiology of autoimmune encephalitis and comparison to infectious causes—Experience from a tertiary center

**DOI:** 10.1002/acn3.52147

**Published:** 2024-07-19

**Authors:** Yahel Segal, Ofer Rotschild, Yair Mina, Gadi Maayan Eshed, Tal Levinson, Yael Paran, Michal Dekel, Ronit Cohen‐Poradosu, Adi Ashkenazi, Itamar Moreno, Orna Aizenstein, Ora Halutz, Yifat Alcalay, Avi Gadoth

**Affiliations:** ^1^ Department of Neurology Tel‐Aviv Medical Center Tel‐Aviv Israel; ^2^ Encephalitis Center Tel‐Aviv Medical Center Tel‐Aviv Israel; ^3^ Infectious Diseases Unit Tel‐Aviv Medical Center Tel‐Aviv Israel; ^4^ Sackler Faculty of Medicine Tel Aviv University Tel Aviv Israel; ^5^ Department of Radiology Tel‐Aviv Medical Center Tel‐Aviv Israel; ^6^ Clinical Microbiology Laboratory Tel‐Aviv Medical Center Tel Aviv Israel; ^7^ Immunology Laboratory Tel Aviv Medical Center Tel Aviv Israel

## Abstract

**Objectives:**

The incidence of autoimmune encephalitis (AIE) has risen in the last decade, yet recent studies are lacking. We compared the epidemiology of autoimmune and infectious encephalitis cases in Tel‐Aviv Sourasky Medical Center (TASMC) between 2010 and 2020.

**Methods:**

All encephalitis cases, aged 18 and above, admitted to TASMC between the years 2010 and 2020 were reviewed for demographic, clinical, laboratory, and imaging data and categorized based on etiology.

**Results:**

Two hundred and twenty‐five patients with encephalitis were identified. The most common identifiable cause was viral (42%), followed by autoimmune encephalitis (35%), bacterial (18%), and fungal/parasitic (5%). The incidence of AIE cases out of the yearly admitted cases increased substantially, from 3.8/100 K in 2010 to 18.8/100 K in 2020. The incidence of viral cases also increased while those of bacterial and fungal/parasitic infections remained stable. Patients with AIE were younger compared to infectious patients (*p‐*value <0.001) and had lower markers of systemic and cerebrospinal fluid inflammation (*p*‐value for all <0.001). Seizures were more common among AIE patients (*p*‐value <0.001), yet one‐year mortality rates were higher among infectious patients (*p*‐value <0.001).

**Interpretation:**

AIE incidence has risen significantly in our institution during the past decade, with current rates comparable to those of all infectious causes combined. Based on this cohort, clinical clues for an autoimmune etiology include a non‐inflammatory cerebrospinal fluid profile, the presence of seizures, and temporal lobe imaging abnormalities (also common in herpetic encephalitis). In light of its rising incidence and the importance of early treatment, AIE should be considered in the differential diagnosis of all encephalitis cases.

## Introduction

Encephalitis, an inflammation of the brain, commonly presenting with cognitive and behavioral changes, seizures, or focal neurological deficits, is an important diagnosis encountered by neurologists. Etiologies include infectious and autoimmune causes.

While infectious causes were previously considered to represent the main volume of these cases,^1^, ^2^ in the past several decades, autoimmune causes are increasingly recognized.^3^, ^4^, ^5^

Autoimmune encephalitis (AIE) refers to a spectrum of conditions including antibody‐associated syndromes, which may target plasma membrane proteins, synaptic proteins, or intraneuronal antigens (viewed as biomarkers of cellular autoimmunity); acute demyelinating encephalomyelitis (ADEM); seronegative AIE; and additional specific syndromes.

A timely diagnosis of these conditions is crucial, as the early initiation of appropriate treatments improves outcomes.^6^, ^7^, ^8^ Furthermore, some of these syndromes may represent a paraneoplastic process, therefore recognition of the neurological syndrome may result in early cancer diagnosis.^7^

The detection rate of AIE has been increasing over the past two decades due to increased awareness of these syndromes among physicians, along with the growing number of identified specific anti‐neuronal antibodies.

In two previous epidemiological studies conducted in the US between 2000 and 2012, AIE cases were recognized in 22% of all encephalitis cases or 17% of cases with an identified cause, substantially less frequently than infectious causes.^1^, ^2^ However, a more recent population‐based study reviewing cases between 1995 and 2015 in Olmsted county, Minnesota reported similar incidence and prevalence rates for autoimmune and infectious causes,^3^ and several studies examining the epidemiology of AIE report a notable increase in cases in the past decade.^9^, ^10^, ^11^ Thus, an updated estimation of the current incidence and prevalence of AIE and its weight compared to IE is lacking.

Herein we describe the incidence, clinical characteristics, and outcomes of AIE patients compared with IE patients in a large tertiary center in Tel‐Aviv, Israel.

## Methods

### Study population

The Tel‐Aviv Sourasky Medical Center (TASMC) is a tertiary center in Israel, which serves a total population of approximately 500,000 people living in the Tel‐Aviv metropolitan area. All patients aged 18 years and above who were hospitalized in our center during 2010–2020 with an infectious/autoimmune/paraneoplastic encephalitis were included. The study was approved by the Institutional Review Board of TASMC.

### Data collection

The electronic database of TASMC was screened for patients admitted between 2010 and 2020 using the following appropriate ICD‐9 diagnoses: encephalitis, encephalopathy, and meningoencephalitis. Additionally, files were extracted of all patients with a pathological CSF profile in this period.

The patients' medical records were reviewed for clinical data, imaging, electroencephalogram reports, and laboratory results including neuronal autoantibodies and immunofluorescence assays (when available).

### Outcome evaluation

Modified Rankin Scale (mRS) was used to assess outcome. Two out of three neurologists (A.G., O.R. and Y.S.) reviewed patients' files and scored the MRS. The average of both reviews was used for the final score.

### Inclusion criteria

**Figure 1:**
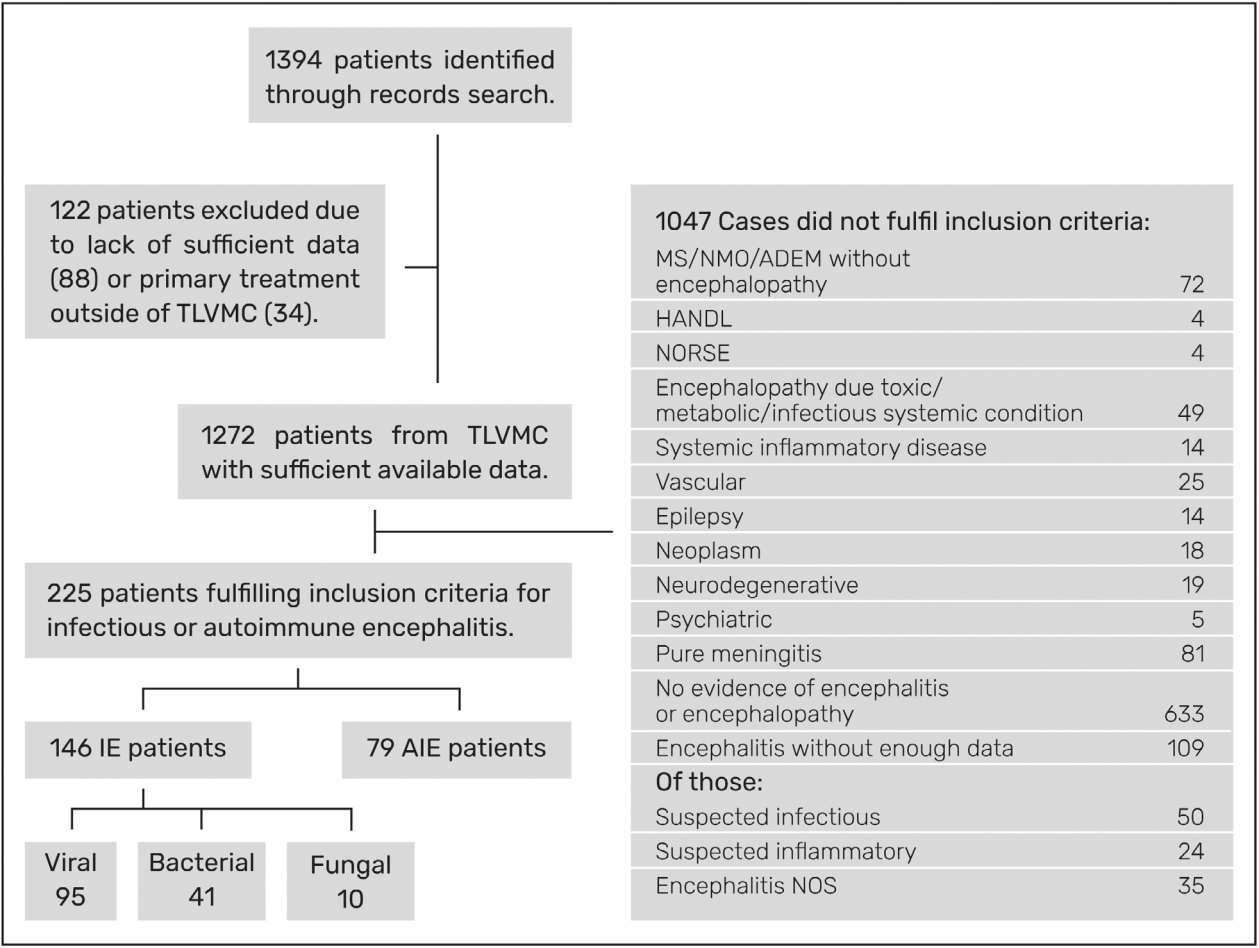
Data inclusion flowchart. AIE, autoimmune encephalitis; IE, infectious encephalitis; ADEM, acute disseminated encephalomyelitis; CAARI, cerebral amyloid angiopathy–related inflammation; HANDL, headache with neurological deficits and elevated CSF lymphocytes; MS, multiple sclerosis; NMO, neuromyelitis optica; NORSE, new‐onset refractory status epilepticus; NOS, not otherwise specified; TASMC, Tel Aviv Sourasky medical center.

For inclusion of patients with AIE we utilized criteria from a position paper by Graus et.al published in 2016, on the approach to AIE (Fig. 1).^12^ These criteria include the diagnosis of seropositive and seronegative AIE, acute disseminated encephalomyelitis, Hashimoto encephalopathy, and Bickerstaff encephalopathy. These criteria require meeting the following conditions: (1) Subacute onset (less than 3 months) of working memory deficits, altered mental status, or psychiatric symptoms; (2) at least one of the following: new focal central nervous system (CNS) finding, unexplained seizures, CSF pleocytosis, and MRI features suggestive of encephalitis; and (3) reasonable exclusion of alternative causes.

Patients suspected of having a paraneoplastic encephalitis had to meet criteria for paraneoplastic encephalitis suggested by Graus et al in 2004.^13^ All AIE suspected cases were reviewed by two out of three qualified neurologists (O.R, A.G and Y.S). We included patients fulfilling criteria for definite and probable AIE, and patients fulfilling criteria for possible AIE in selected cases that were highly suggestive of AIE. Only patients accepted by both reviewers were included. A detailed report of all AIE cases including possible autoimmune can be found in the supplementary data.

For inclusion of patients with IE, we applied the 2013 diagnostic criteria for possible or probable encephalitis of presumed infectious etiology,^14^ requiring an altered mental status lasting at least 24 hours, along with two of five minor criteria. Only cases with an identified pathogen were included. All suspected cases of infectious encephalitis were reviewed by three qualified neurologists (G.M, A.G and Y.S) and a qualified infectious diseases specialist (T.L).

### Laboratory testing

Serum and cerebrospinal fluid (CSF) from patients suspected for AIE were tested for the presence of specific cell surface anti‐neural autoantibodies using a cell based assay (CBA) (Euroimmun, Lubeck, Germany) that include anti‐NMDA, anti‐GABA‐B, anti‐AMPA, anti‐LGI1, and anti‐CASPR2 antibodies, or for the presence of paraneoplastic antibodies (using immunoblot (Euroimmun) that include ANNA‐1 (Hu), ANNA‐2 (Ri), PCA‐1 (Yo), anti‐amphiphysin, anti‐CV2/CRMP5, anti‐SOX1, Anti‐Ma2, anti‐Titin, anti‐Recoverin). Indirect immunofluorescence assay (IFA) constructed from adult mouse tissues: cerebellum, midbrain, cerebellar cortex, hippocampus, kidney, and gut, was also performed as previously described.^15^

The presence of anti‐MOG antibodies was determined by a fixed cell‐based assay (EUROIMMUN, Lubeck, Germany).

The presence of anti‐ganglioside antibodies was determined by immunoblot technique, using EUROLine kits (EUROIMMUN, Lubeck, Germany).

Thyroglobulin (TG) and microsomal thyroid peroxidase (TPO) antibodies were analyzed using the automated Alegria® system (ORGENTEC Diagnostika, Germany).

Antibodies against glutamic acid decarboxylase (GAD) were detected using Elisa (RSR, UK).

Infectious pathogens were detected with serum and/or cerebrospinal fluid (CSF) cultures, specific polymerase chain reaction (PCR) testing (Herpes Simplex Virus (HSV) 1&2, Enterovirus, Varicella Zoster Virus (VZV), Human Herpes Virus (HHV) 6, Epstein–Barr Virus (EBV), Toxoplasma), serological testing (West Nile Virus (WNV), *Coxiella burnetii* (Q‐FEVER), Human Immunodeficiency Virus (HIV)), specific antigen testing (Cryptococcus), and the Biofire Film Array panel (testing for Escherichia coli, *Haemophilus influenzae*, *Listeria monocytogenes, Neisseria meningitidis, Streptococcus agalactiae, Streptococcus pneumoniae*, Cytomegalovirus, Enterovirus, HSV‐1&2, HHV‐6, HPeV, VZV, and Cryptococcus).

### Statistical analysis

The statistical analysis encompassed several key components designed to investigate various aspects of encephalitis, with a specific focus on comparing autoimmune encephalitis (AIE) with infectious encephalitis (IE), and further dissecting AIE by comparing it with bacterial, viral, and fungal subcategories.

Continuous variables were summarized using the median and interquartile range (IQR) then compared between groups. When comparing two groups (AIE vs. IE), the Mann–Whitney test was employed. For comparisons involving four groups (AIE, bacterial, viral, and fungal), an initial Kruskal–Wallis test was conducted to assess overall differences. Subsequently, Dunn's test was utilized for pairwise comparisons.

Categorical variables were summarized using counts and percentages to provide insights into the distribution of categories within each group. Group comparisons for categorical variables were accomplished using the chi‐square test to examine associations or differences.

To delve deeper into factors related to mortality, a logistic regression model was applied. This model incorporated variables such as encephalitis type, gender, age, presence of seizures and the modified Rankin Scale (mRS) score before the event as predictors.

A two‐tailed *p*‐value less than 0.05 was considered statistically significant.

The statistical analyses were carried out using R‐4.3.1 (R Foundation for Statistical Computing, Vienna, Austria).

## Results

Between 2010 and 2020 a total of 225 patients were diagnosed in our center with infectious or autoimmune encephalitis (Fig. 1).

Of those, 42.2% (95 patients) were diagnosed with viral encephalitis, 35.1% (79 patients) with AIE, 18.2% (41 patients) with bacterial encephalitis and about 4% (10 patients) with fungal or parasitic encephalitis.

**Figure 2:**
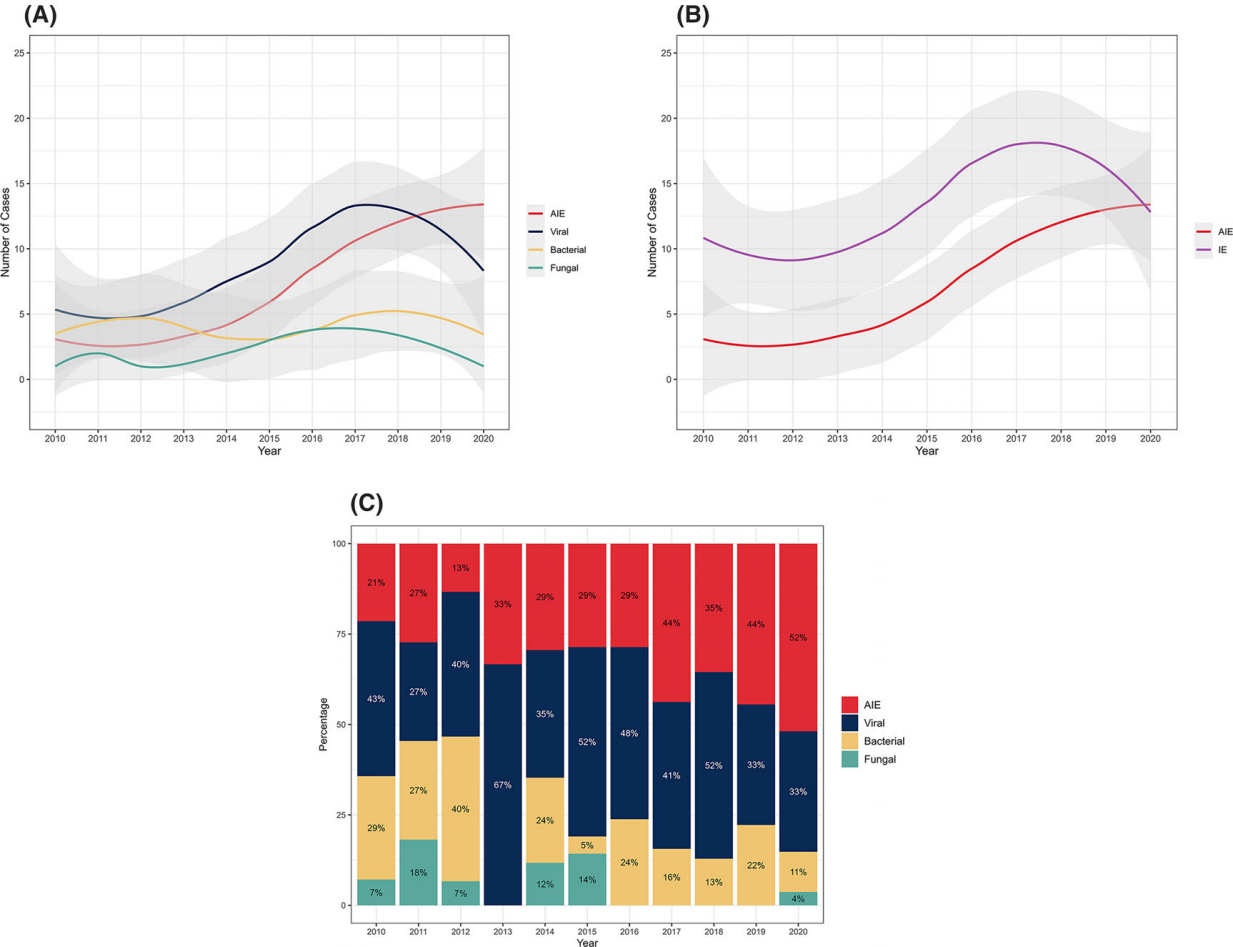
Number of encephalitis cases per year by etiology. (A) Shows each sub‐group individually. (B) Shows AIE compared to all IE causes combined. (C) Yearly percentages of each sub‐group out of all annual cases. AIE, autoimmune encephalitis; IE, infectious encephalitis.

The annual incidence of AIE in our institution showed a notable increase in absolute numbers as well as in the proportion out of all cases (Fig. 2). The annual incidence of AIE cases increased more than fourfold, rising from about 3.8 cases per 100 K admitted patients in 2010 to 18.8 per 100 K admitted patients in 2020.

In terms of proportion of total encephalitis cases, the average 3‐year rate of AIE cases nearly doubled from 22.4% of all encephalitis cases for the years 2010–2013 to 43.6% for the years 2017–2020.

The average incidence of viral encephalitis showed a significant increase as well, with an initial incidence of 7.7 cases per 100 K admitted patients, peaking at 20 cases per 100 K admitted patients in 2018, then stabilizing at an average of approximately 11.5 cases per 100 K admitted patients in the following 2 years. In contrast, the rates of bacterial encephalitis seemed to remain relatively steady over the years, peaking twice in 2012 and 2019 with six yearly cases, which corresponded to 7 cases per 100 K admitted patients in those years. Cases of fungal/parasitic encephalitis showed a similar trend, though the low absolute numbers of these cases limit the ability to draw valid conclusions.

### Epidemiological features of AIE versus IE

Patients with AIE presented at a younger age of onset compared to all infectious causes combined (median age 59 vs. 67 years, *p*‐value <0.001) (Table 1). When examining infectious causes individually, patients with fungal encephalitis appeared to present at a younger age, significantly younger than patients with bacterial or viral encephalitis (median age 36 vs. 66 and 70 years accordingly, *p*‐value = 0.006).

**Table 1:** Demographic characteristics of AIE versus IE.

	AIE	Bacterial	Fungal	Viral	Total	*p*‐value	*p*‐value for AIE versus IE
Number of patients	79	41	10	95	225		
Age at presentation							
Median (Q1, Q3) Min–Max	59.5 (42, 70) 17–88	66 (61, 77) 35–91	36.5 (31, 44.8) 25–67	70 (58.5, 81.5) 25–94	65 (47, 76) 17–94	<1e‐04	0.0006
Female sex (%)	38 (48.1%)	22 (53.7%)	3 (30.0%)	36 (37.9%)	99 (44.0%)	0.2349	0.3998
mRS prior							
≤2 (%)	79 (100.0%)	32 (78.0%)	8 (80.0%)	79 (83.2%)	198 (88.0%)	0.0005	<1e‐04
>2 (%)	0 (0.0%)	9 (22.0%)	2 (20.0%)	16 (16.8%)	27 (12.0%)		

AIE, autoimmune encephalitis; IE, infectious encephalitis; mRS, modified Rankin Score.

Patients with AIE appear to represent a healthier population, as demonstrated by a lower mRS prior to admission. All AIE had mRS ≤2 as compared to 81.5% among IE (*p*‐value <0.001).

### Clinical features of AIE versus IE

Temperature at admission was lower among AIE patients compared to IE patients (median temperature 36.8 vs. 37.8 degrees Celsius, *p*‐value <0.001) (Table 2). When examining individual infectious causes, bacterial patients clearly stand out with a median temperature of 39. Though AIE patients presented with temperatures significantly lower than each of the infectious causes, the 25th–75th interquartile range of AIE overlaps that of viral patients [36.5–37] versus [36.8–38.2].

**Table 2:** Clinical characteristics of AIE versus IE.

	AIE	IE combined	Bacterial	Fungal	Viral	Total	*p*‐value	*p*‐value for AIE versus IE
Temperature at admission, *N* Median (Q1, Q3)	72 36.8 (36.5, 37.0)	134 37.8 (36.9, 38.8)	37 39.0 (38.3, 39.2)	7 37.2 (37.2, 38.0)	90 37.5 (36.8, 38.2)	206 37.1 (36.7, 38.3)	<1e‐04	<1e‐04
CRP levels, *N* Median (Q1, Q3)	73 2.0 (0.0, 9.0)	132 20.1 (3.9, 75.3)	40 131.0 (28.8, 193.2)	6 9.8 (5.5, 18.8)	86 10.5 (1.2, 25.4)	205 9.7 (1.0, 31.2)	<1e‐04	<1e‐04
WBC in serum, *N* Median (Q1, Q3)	76 9.4 (7.0, 11.1)	145 10.8 (7.9, 13.6)	40 15.4 (11.8, 20.2)	10 5.9 (4.1, 12.5)	95 9.6 (7.8, 11.9)	221 9.8 (7.7, 12.9)	<1e‐04	0.0267
CSF characteristics								
WBC, *N* Median (Q1, Q3)	74 4.0 (0.0, 20.8)	131 183.0 (46.0, 531.5)	39 764.0 (296.5, 1828.0)	6 6.5 (2.0, 17.0)	86 86.0 (30.2, 298.8)	205 68.0 (5.0, 330.0)	<1e‐04	<1e‐04
Protein, *N* Median (Q1, Q3)	74 53.0 (37.0, 92.8)	132 130.5 (67.5, 228.5)	39 348.0 (205.5, 403.5)	7 66.0 (42.5, 110.5)	86 89.0 (57.2, 150.5)	206 87.5 (49.2, 180.8)	<1e‐04	<1e‐04
Glucose, *N* Median (Q1, Q3)	71 57.0 (50.5, 65.0)	131 53.0 (33.0, 72.5)	39 21.0 (2.5, 37.0)	8 69.5 (41.5, 90.0)	84 58.5 (48.0, 75.2)	202 55.5 (46.0, 69.0)	<1e‐04	0.0946
Oligoclonal bands Intrathecal synthesis (%)	18/56 (32.1%)	1/5 (20.0%)	0 0	0 0	1/5 (20.0%)	19/61 (31.1%)		1
Seizures Present (%)	28/79 (35.4%)	21/145 (14.5%)	5/41 (12.2%)	3/9 (33.3%)	13/95 (13.7%)	49/224 (21.9%)	0.0015	0.0006
1‐year mortality Deaths (%)	4/79 (5.1%)	34/124 (27.4%)	10/39 (25.6%)	3/9 (33.3%)	21/76 (27.6%)	38/203 (18.7%)	0.0005	<1e‐04
Follow‐up duration (months), *N* Median (Q1, Q3)	78 31.5 (17.0, 48.2)	142 1.4 (0.0, 11.0)	39 2.0 (0.4, 6.5)	9 2.0 (0.8, 4.0)	94 1.0 (0.0, 26.4)	220 9.0 (0.2, 36.0)	<1e‐04	<1e‐04

AIE, autoimmune encephalitis; CRP, C‐reactive protein; CSF, cerebrospinal fluid; IE, infectious encephalitis; mRS, modified Rankin Score; WBC, white blood cells.

Serum leukocyte counts were lower among AIE patients compared to IE patients (median 10.0 10e3/μL vs. 11.7 10e3/μL, *p*‐value = 0.027). The difference was again more pronounced when comparing bacterial patients to all other causes. Of the three patients with AIE who presented with extreme leukocytosis (≥20), one was a patient with anti‐GAD AIE admitted with severe DKA, another, who was later diagnosed with ADEM, was admitted following a motorcycle accident, and the third, diagnosed with possible seronegative AIE, was admitted following a course of IVMP in another medical facility. All three had normal or near normal levels of Serum C‐reactive protein (CRP).

As may be expected, CRP levels were significantly elevated among infectious patients compared to AIE patients (median 20.1 mg/L vs. 2.0 mg/L, *p*‐value <0.001), and this was apparent also when comparing AIE individually to bacterial or viral causes. However, the 25‐75th interquartile range of AIE overlaps that of viral patients. Unsurprisingly, bacterial patients showed the highest values, significantly higher than viral patients by 12.5 fold.

CSF leukocyte counts were significantly lower among AIE patients when compared to infectious causes and individually to viral or bacterial encephalitis. Counts were similar between AIE and fungal encephalitis (median values were 4 cells/μL for AIE, 764 cells/μL for bacterial, 86 cells/μL for viral, and 6.5 cells/μL for fungal). Notably, among AIE patients, only five patients presented with CSF leukocyte counts above 200 cells/μL. Two were ADEM patients, one was diagnosed with anti‐GFAP AIE, and two were seronegative.

Normal CSF leukocyte counts were noted in 54% of AIE cases (40/74) compared to 8% of IE cases (11/131, *p*‐value <0.001.).

CSF protein values were also significantly lower among AIE patients (median was 53 mg/dL for AIE versus 130 mg/dL for infectious causes, *p*‐value <0.001). Though individual comparison showed a significant difference between AIE and viral encephalitis, the 25–75th interquartile range of the two again overlaps.

Normal CSF protein levels were noted in 42% of AIE cases (31/73) compared to 10% of IE cases (14/132, *p*‐value <0.001).

CSF glucose levels were notably lower in bacterial encephalitis compared to AIE or viral encephalitis (21, 57, and 58.5 respectively).

A completely normal CSF profile (normal values of leukocytes and protein as well as negative oligoclonal bands) was noted in 24 AIE patients (33%) compared to three IE patients (~2%).

Magnetic resonance imaging (MRI) data was available for 180/225 patients. Among those, the location of imaging findings, when observed, did not vary significantly between causes. Most patients in all groups combined presented with normal imaging (63%), and this was also true when examining the AIE (59%) and viral (69%) groups separately. Abnormal findings were most common in the temporal areas (27 patients, 16 AIE and 11 viral). Notably, out of 11 viral patients with temporal involvement, 8 (73%) had HSV1.

With regards to clinical presentation, seizures were recorded more frequently among AIE patients compared to infectious causes (35.4% vs. 14.5% *p*‐value <0.001).

### Follow‐up and outcomes of AIE versus IE

Median follow up duration was longer for AIE patients compared to infectious causes (31.5 months vs. 1.4 months, *p*‐value <0.001).

One‐year mortality rates were significantly higher among infectious causes compared to AIE (27.4% vs. 5.1%, *p*‐value <0.001).

**Figure 3:**
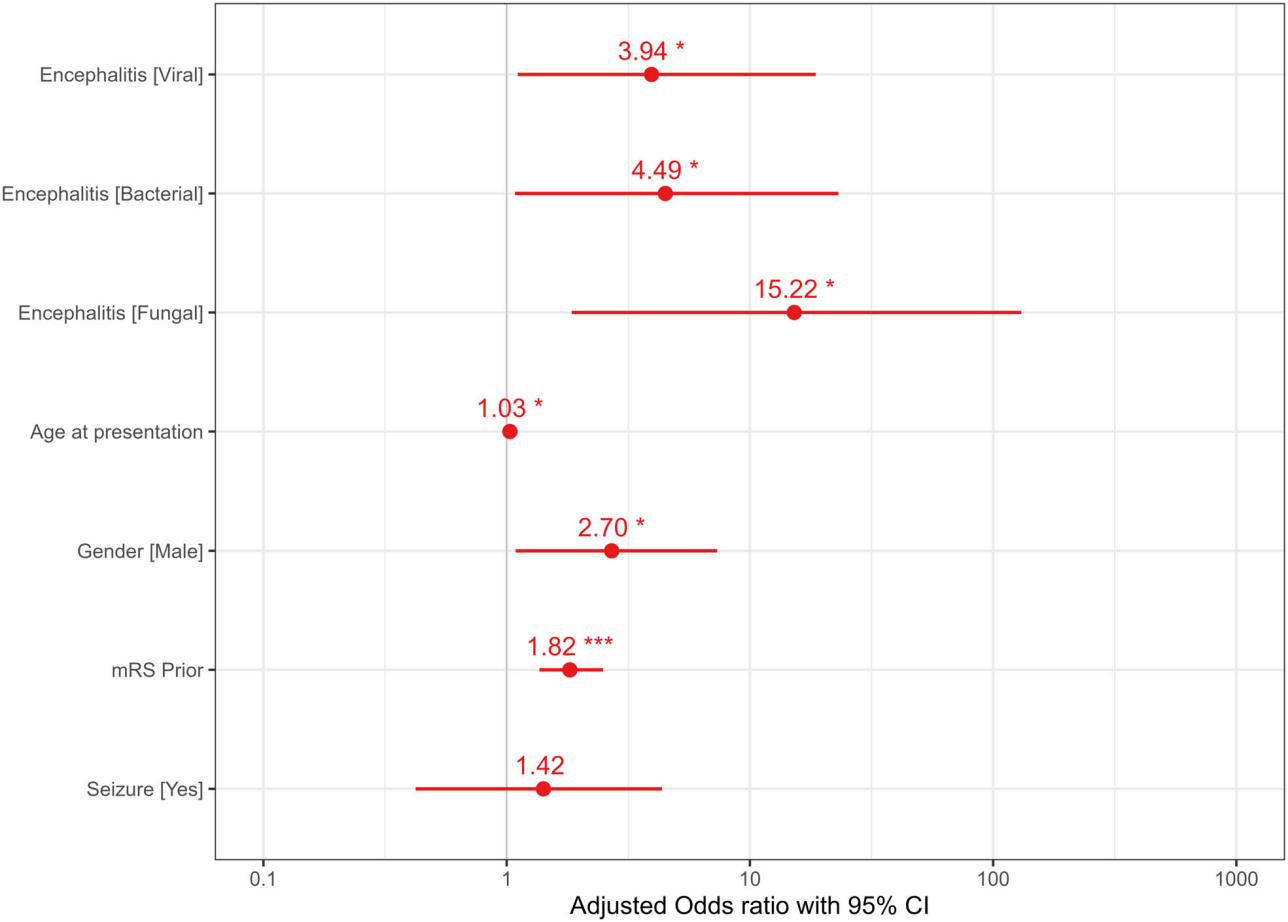
Logistic regression of factors affecting 1‐year mortality. CI, confidence interval; mRS, modified Rankin Score; *p < 0.05; **p < 0.01; ***p < 0.001.

Logistic regression analysis of multiple variables affecting 1‐year mortality (Fig. 3) showed each of the infectious encephalitis types to independently carry a significant increased odds on 1‐year mortality (OR = 3.94, CI 1.11–18.66; OR = 4.49, CI 1.08–23.13; 15.22 CI 1.85–130.53 for viral, bacterial, and fungal, respectively). Additional factors carrying effect on 1‐year mortality were mRS prior to disease (OR = 1.82, CI 1.36–2.49) and male gender (OR = 2.70, CI 1.09–7.34). Of note, age was not found to carry an independent effect on 1‐year mortality (OR = 1.03, CI 1–1.06, *p*‐value = 0.037).

### IE patients

**Figure 4:**
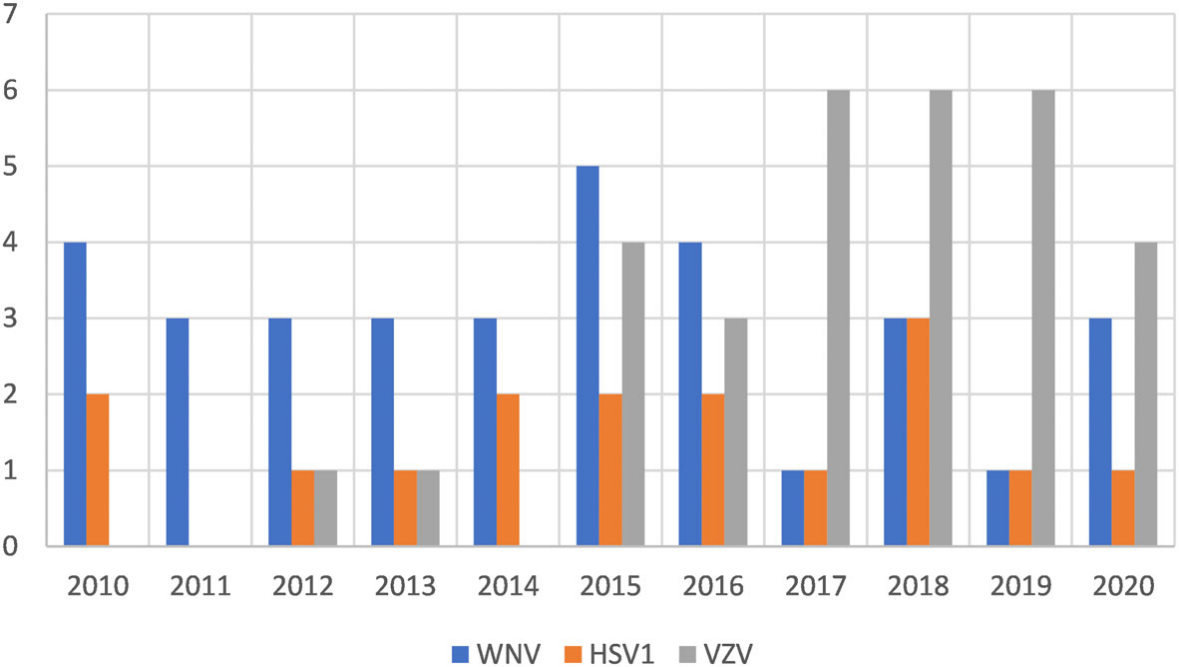
Chief viral causes of infectious encephalitis by year. HSV1, Herpes Simplex 1 Virus; VZV, Varicella Zoster Virus; WNV, West Nile Virus.

The most common infectious cause was viral, showing a significant increase in incidence during 2010–2020. The most common etiologies (Fig. 4) were West Nile Virus (WNV) with 33 cases (34.7% of viral IE cases), Varicella‐Zoster Virus (VZV) with 31 cases (32.6% of viral cases), and Herpes Simplex Virus 1 (HSV1) with 16 cases (16.8% of viral cases). Of these three most common causes, patients with HSV1 presented at a younger median age, while WNV and VZV showed a similar median age of presentation in the 8^th^ decade (65, 73, and 76 years old respectively). A distinct rise in the number of VZV cases was noted from 2016 and on, appearing to be the driver of the overall increase in viral cases.

Out of 21 mortality cases within the viral encephalitis group—45% (9 patients) had WNV, 19% (4 patients) had VZV, and 15% (3 patients) had HSV1, and additional causes were HSV2, HHV‐6, and EBV. All deaths reported occurred in the first year.

Of the bacterial cases, (23/41) 56.6% had infection with *Streptococcus pneumoniae*, and 29% (12 patients) with *Listeria monocytogenes* (the youngest patient was 57 years old). Additional rare etiologies included *Neisseria meningitidis*, *Escherichia coli*, Rickettsia, *Klebsiella pneumoniae*, and *Streptococcus pyogenes*.

One year mortality rate among bacterial cases was 24% (10/41). The infectious agent in these cases was most commonly *Listeria monocytogenes* (60% of deaths and 50% of all Listeria cases) followed by *Streptococcus pneumoniae* (40% of deaths and 17% of all Pneumococcal cases).

Patients classified as having fungal/parasitic encephalitis were most commonly diagnosed with Toxoplasma (7/10); all were HIV positive. Additional etiologies included Cryptococcal infection and invasive Aspergillus, both in immunocompromised patients.

Of all IE cases, 11% (16/146) were immunosuppressed. Pathogens detected among immunosuppressed patients included *Listeria monocytogenes* (6 cases), HHV‐6 (3 cases), WNV, EBV, (both with 2 cases), VZV, HSV‐1, and HSV‐2 (with one case each). Two additional patients were diagnosed with HIV encephalitis with low CD4 counts and were immunosuppressed due to their viral illness.

### AIE patients

A total of 79 AIE patients were diagnosed and treated in the Tel‐Aviv medical center between 2010 and 2020.

Among the AIE cases, 43 were seropositive (five of whom were with an unclassified positive IFA), and 22 were seronegative. Additional diagnoses included ADEM, Hashimoto's encephalopathy and Bickerstaff encephalitis (Table 3).

**Table 3:** AIE cases by type.

Type	Number	% of AIE
Seronegative	22	27.8%
Anti‐LGI1	17	21.5%
Anti‐NMDA	8	10.1%
ADEM	7	8.9%
Undefined antibody on Immunofluorescence assay	5	6.3%
Hashimoto	5	6.3%
Anti‐GAD65	4	5.1%
Anti‐CASPR2	2	2.5%
Bickerstaff encephalitis	2	2.5%
Anti‐Ma‐2	2	2.5%
ANNA2/Ri	1	1.3%
Anti‐GFAP	1	1.3%
Anti‐GABA‐B	1	1.3%
PCA‐2/MAP1B	1	1.3%
ANNA1/Hu	1	1.3%
Total	79	

ANNA, anti‐neuronal nuclear antibody; ADEM, acute disseminated encephalomyelitis; CASPR2, contactin‐associated protein‐like 2; GABABR, gamma‐aminobutyric acid B receptors; GAD65, glutamic acid decarboxylase 65‐kilodalton isoform; GFAP, Glial fibrillary acidic protein; LGI1, leucine‐rich glioma inactivated 1; MAP1B, microtubule associated protein 1B; NMDAR, N‐methyl‐D‐aspartate receptor; PCA, Purkinje cell antibody.

Of the seronegative AIE patients, three met the criteria for definite limbic or paraneoplastic AIE, two met the criteria for probable AIE and seventeen met the criteria for possible AIE.

A specific anti‐neural antibody was recognized in 38 patients (51%), most commonly anti‐LGI1 (17 patients) followed by anti‐NMDA (8 patients). Approximately 7% of patients had a positive immunofluorescence assay showing a distinct yet unrecognized pattern and fulfilled criteria for possible, probable, or definite AIE, categorized here as AIE with an unclassified pattern. An additional prominent diagnosis was ADEM, reported in 9% of cases.

## Discussion

During the decade reviewed, the incidence of AIE increased considerably, with rates more than quadrupling between 2010 and 2020, and a correlating increase in the proportion of AIE cases out of all encephalitis cases. The incidence of viral IE showed a similar, perhaps more moderate increase, while bacterial cases mildly decreased. These findings are in accordance with previous studies reporting of viral IE as the leading cause of encephalitis,^8^ as well as the trend appreciated in recent studies of the increasing incidence of AIE in recent decades.^3^, ^9^, ^10^, ^11^, ^16^ There may be several possible explanations for this epidemiological shift.

AIE represents a relatively novel set of syndromes, and as such, some of these syndromes were initially reported and defined during the time‐period examined in our study, while several others gained from recent developments of improved diagnostic technics; for example, the exact antigen for anti‐LGI1 was discovered in 2010^17^ ^(p1),^ ^18^ with CBA becoming prevalent during the following several years, the syndrome of anti‐GFAP meningoencephalitis was initially reported merely in 2016^19^, and CBAs for MOG antibodies were optimized during the second half of the decade in question.^20^ Thus, the rate of diagnosis of AIE may still be on the rise as new tests continue to emerge and awareness for the newly discovered syndromes gradually increases.

The contribution of such innovations in AIE research to the increase in AIE incidence is supported by parallel reports demonstrating a similar rise in AIE incidence in various populations in Europe, the US and China^3^, ^10^, ^11^, ^21^ in the past decades. Furthermore, evidence suggests that in the younger population the frequency of autoimmune causes may surpass that of infectious etiologies.^22^, ^23^

An additional significant factor unique to our study population, was the establishment of an Encephalitis center in the Tel Aviv medical center in 2017, which includes an autoimmune encephalitis service and laboratory. With the establishment of this center, IFA testing was introduced as a routinely accessible in‐house test. This provided the opportunity to recognize antibody patterns which are not included in the routinely used paraneoplastic and autoimmune panels in our institution, resulting in improved diagnostic accuracy, as we have recently reported.^24^ In our current cohort, IFA enabled the diagnosis of two AIE patients carrying antibodies not included in our routine CBA kits (GFAP and PCA2/MAP1B), and five additional cases of AIE presenting with an unclassified positive IFA pattern.

A parallel increase in incidence was observed in viral IE cases in our cohort. This rise was contrary to a reported reduction in cases of viral CNS infections in China in parallel years.^25^ Yet this Chinese surveillance study examined meningitis as well as encephalitis cases, and related only to four possible viral etiologies, while a specific pathogen was not identified in 70% of cases. Thus, some of the unrecognized cases may represent viral etiologies which were not tested, such as varicella zoster, which was a common cause of viral IE in our cohort.

In fact, examination of the specific etiologies of viral IE suggests the most prominent driver of the increased incidence is a rise in the number of VZV cases. This is in line with previous reports of a global increase in VZV cases in the past several decades,^26^, ^27^ pointing to VZV as the most common cause of viral encephalitis among elderly patients, followed by HSV1.^28^ Several hypotheses have been raised regarding this increase in rates of VZV infection. Among those were an increased awareness among medical staff, an increase in prevalence of immunocompromised individuals, and an effect of environmental and climate changes, yet none were corroborated and some were proven inaccurate.

With regards to our specific cohort, an additional possible contribution to the increased incidence of viral cases could perhaps be attributed to the incorporation of specific in‐house PCR testing for VZV and HSV in 2014. Indeed, the number of VZV cases seems to triple between 2013 and 2015 and remains high thereafter. Yet the lack of a parallel rise in HSV cases suggests that increased testing is not the only factor at play.

Apart from the noteworthy shift in encephalitis etiologies, we examined the main epidemiological and clinical characteristics of each etiological group in search of defining features which can aid in the differential diagnosis of an encephalitic patient at presentation.

As may be expected, AIE patients were younger and healthier at presentation than infectious patients, probably due to the known association of older age and comorbidities with certain infectious causes, while autoimmune diseases in general tend to occur in a younger population with an active immune system. This is despite the fact that certain AIE syndromes have an average age of onset in the seventh decade, with several reports of diagnosis in the ninth decade.^29^, ^30^

In terms of diagnostic features at presentation, AIE patients presented in general with lower systemic inflammatory markers and lower temperatures at admission compared to IE patients. Variations in these parameters were all significantly more pronounced when comparing bacterial IE to AIE. However, the utility of these differences may be limited in the clinical setting where often a differentiation is required between AIE and viral IE, as the 25th–75th interquartile range of these parameters overlaps between the two etiologies.

Examination of CSF inflammatory markers showed a similar pattern, with AIE patients presenting with lower protein levels and lower CSF cell counts. In fact, more than 50% of AIE patients presented with normal CSF WBC counts, and ~ 40% presented with normal protein levels. The parallel rate of IE patients with normal CSF WBC counts or normal CSF protein levels was, as expected, substantially lower at ~9% for each parameter. A completely normal CSF profile (normal values of leukocytes and protein as well as negative oligoclonal bands) was noted in 24 AIE patients (33%) compared to merely three IE patients (2%). Thus, it seems that a normal CSF profile substantially increases the likelihood of an autoimmune etiology compared to an infectious cause. However, we were unable to provide a threshold value of protein level or pleocytosis to confidently distinguish AIE from viral IE. Similar findings were reported in a recent study looking for laboratory biomarkers associated with AIE versus IE.^31^

Imaging data proved less informative, as most of the patients in both viral and AIE groups presented with normal imaging. As may be expected, the most common location for imaging abnormality was in the temporal lobes, and these patients belonged either to the AIE or the viral groups (72% of these viral cases were HSV1 positive). This may be explained by the predilection to temporal location of many of the common AIE syndromes (presenting as limbic encephalitis) as well as of herpetic encephalitis, one of the common viral causes of encephalitis. Thus, an initial presentation with abnormal temporal lobe imaging may provide a clue to an either autoimmune or herpetic diagnosis.

The last clinical feature examined was seizures, which were significantly more common among AIE patients compared to infectious patients. The presence of seizures showed a trend but failed to show a significant effect on 1‐year mortality rates.

Nonetheless, in terms of prognosis, AIE patients showed lower mortality within 1 year, in compliance with previous findings.^32^ A possible explanation for this could be that AIE patients were on average younger and healthier at presentation, as suggested by the significant effect of mRS prior to disease on mortality rates.

We further examined the specific characteristics of each autoimmune cause. While our description of the two most common AIE syndromes of anti‐LGI1 and anti‐NMDA is in line with numerous previous descriptions, we describe two intriguing and less frequently addressed categories—unclassified and seronegative AIE.

Our 22 seronegative AIE patients amassed to form the largest classification among AIE patients, representing 28% of all AIE cases. This rate is slightly higher than the previous epidemiological study from Mayo,^3^ while two more recent studies examining seronegative AIE related to substantially higher rates of seronegative cases, approximately 50% of all AIE cases examined.^33^, ^34^

Our seronegative patients presented most commonly with cognitive decline, confusion, and behavioral changes, showed an inflammatory CSF profile and a good response to treatment, with 68% showing a favorable outcome (mRS 0–2 on follow‐up). These findings vary from those of two recently published cohorts of seronegative patients from China and Germany, where a favorable outcome was reported in 56% and 92% of patients accordingly.^33^, ^34^

As to our patients with unclassified IFA patterns, they appear to represent a heterogenous group in terms of symptoms and imaging findings, yet most showed an inflammatory CSF profile. Remarkably, 3/5 did not respond to immunomodulatory treatment (one was a paraneoplastic case that improved following tumor resection).

Our study has some limitations. As it is a retrospective observational longitudinal study, cases which were not documented properly, or in which encephalitis was not considered in the differential diagnosis at the time of admission, are missing. Furthermore, there is a substantial variation in follow‐up duration between the different groups, perhaps partially attributable to the more chronic nature of AIE compared to IE, yet this naturally causes variations in data resolution and limited our ability to compare long term outcome.

Additionally, our clinical laboratory does not perform testing for several rare yet well‐established antibodies such as anti‐glycine, anti‐mGluR1and anti‐GABA‐A. While IFA provides opportunity to detect some of these, the absence of specific testing may have resulted in underdiagnosis of these conditions.

In terms of inclusion criteria, we did not include cases under 18 years old thus our findings may not be applicable to the pediatric population. A recent study of encephalitis epidemiology in this population reported similar rates of AIE and IE, with high rates of WNV similar to ours, yet the specific etiologies of AIE differed, as may be expected considering known variations in median age of onset of these diseases.^16^ Furthermore, the decision to include only cases where a distinct pathogen was detected may have caused an underestimation of IE cases, related to restrictions in our center's available infectious tests. On the other hand, AIE inclusion criteria allowed for the inclusion of seronegative cases (as accepted in the field). We note that this cohort includes a substantial number of seronegative cases. In such cases confirming the diagnosis of AIE is always more complex and the possibility of misdiagnosis remains a challenge in this group of patients. Therefore we aimed to strictly adhere to the pointers suggested in the two cardinal recent publications regarding AIE misdiagnosis^35^ and pitfall in the diagnosis of seronegative AIE.^36^ All seronegative cases in our cohort fulfilled the three minimal requirements for possible AIE and only cases where extensive workup was done to rule out alternative diagnoses were included. Moreover, all our seronegative patients were tested in serum and CSF for AIE related autoantibodies, and IFA was performed in most of them. None had isolated psychiatric symptoms, and more than half had a pathologic EEG. Most of our patients responded to immunotherapy, further supporting the diagnosis. Even so, the possibility of misdiagnosis remains.

In this aspect, it is worth mentioning metagenomic next generation sequencing^37^ which is gradually being incorporated in the diagnostic workup of many facilities including our own, and may improve detection of infectious pathogens among seronegative cases.

Finally, our cohort represents the epidemiology of infectious and autoimmune encephalitis in Israel, as may be shown by the high incidence of WNV cases compared to Tic Born encephalitis cases. Previous evidence suggests such geographic and ethnic variations affect not only the variety of infectious pathogens but also the epidemiology of autoimmune encephalitis,^10^, ^11^, ^21^ partially due to genetic variations between populations.^38^, ^39^, ^40^ Thus, extrapolations from our study to other populations and geographic locations should be done with caution.

However, even considering these limitations, this study provides an updated robust assessment of the epidemiology of autoimmune and infectious encephalitis in a large population in Israel.

### Conclusion

Our findings point to a substantial increase in the rates of autoimmune encephalitis during the past decade, with recent rates comparable to those of all infectious causes combined. We further noted an increase in the rates of viral encephalitis. Possible explanations for these trends include the increased awareness and improved diagnostics in the field of AIE, along with a global rise in the incidence of VZV CNS infections.

We found AIE patients to present at a younger age and healthier conditions compared to IE patients, with substantially less systemic inflammatory markers, and a less inflammatory CSF profile. While these parameters show significant variations between AIE and IE as a whole, we found there are no clear cutoff values enabling simple differentiation between viral and autoimmune encephalitis.

Due to differences in follow‐up duration, we could not compare long term outcome between IE and AIE patients. We do, however, show considerably higher mortality rates at 1 year among IE patients, and that each of the infectious encephalitis types, independently, carry a significant effect on 1‐year mortality.

We believe that familiarity with the chief characteristics described for each etiology, as well as the general epidemiological shifts observed in our cohort, can directly aid neurologists improve the diagnostic algorithms of patients presenting with new onset encephalitis. AIE should be kept high in the differential diagnosis considering its comparable incidence to infectious encephalitis and the importance of early treatment.

## Author contributions

Y.S: Conception and design of the study, acquisition and analysis of data, drafting a significant portion of the manuscript and figures. O.R: Conception and design of the study, acquisition and analysis of data, drafting a significant portion of the manuscript and figures. Y.M: Acquisition and analysis of data. G. M.E: Acquisition and analysis of data. T.L: Acquisition and analysis of data. Y.P: Acquisition and analysis of data. M.D: Acquisition and analysis of data. R.C.P: Acquisition and analysis of data. A.A: Acquisition and analysis of data. I.M: Acquisition and analysis of data. O.A: Acquisition and analysis of data. O.H: Acquisition and analysis of data. Y.A: Acquisition and analysis of data. A.G: Conception and design of the study, acquisition and analysis of data, drafting a significant portion of the manuscript.

## Funding Information

No funding information provided.

## Conflicts of Interest

The authors have no conflicts of interest.

## Supporting information


Data S1.

## Data Availability

Data are available in supplementary materials; further data are available upon request from the authors.
