# Supplementary material for: Epidemiology of autoimmune encephalitis and comparison to infectious causes—Experience from a tertiary center
Source: Ann Clin Transl Neurol. 2024 Jul 19;11(9):2337–49. doi: 10.1002/acn3.52147 (PMC11537142; doi:10.1002/acn3.52147)
Supplement: Supplementary file 1 — Data S1. [file ACN3-11-2337-s001.docx]

**Supplementary data**

Clinical features and outcomes of various AIE etiologies

**LGI1**

Patients with anti-LGI1 antibody encephalitis were more frequently males, with a median age of 64 years, in accordance with previous reports. The most common presenting symptoms were seizures, followed by cognitive deficits and behavioral changes. Most patients presented with a noninflammatory CSF profile, with no pleocytosis and normal or mildly elevated protein levels. Hyponatremia was a prominent feature in this group. None had systemic inflammatory markers. Imaging was unremarkable or with non-specific or unrelated changes in most. Unlike previous reports, most patients had an abnormal initial EEG.

All the patients were treated with immunomodulatory therapy, and all showed clinical response, mostly with an overall good clinical outcome, though readmissions were common, reported in 8/17.

**NMDA**

Mirroring the anti LGI1 encephalitis patients, patients with anti-NMDA antibody encephalitis, presented at a younger age (median age was 33.5) and were predominantly females (75%). The most common presenting symptoms were cognitive decline, memory disturbances and encephalopathy, reported in some combination in all the patients, accompanied by seizures in 5 patients (62%). Of note, only one patient presented with overt psychosis.

Lumbar puncture was pathologic in most patients (75% 6/8); four (50%) showed pleocytosis (median 5.5, range 5-250 cells/µL, normal range 0-5 cells/µL), three (38%) showed increased protein content (58-244 mg/dL, normal range 12.0-45.0 mg/dL), and three tested positive for intrathecal synthesis of oligoclonal bands. None had serum electrolyte disturbances. Brain imaging, in contrast to most series, was frequently abnormal (62%, 5/8), mainly showing hyperintensities of the temporal lobe.

All the patients were treated with steroids acutely, and 75% were treated additionally with either IVIG or plasmapheresis. 62% required second line therapy with rituximab. Following treatment, substantial clinical response was documented in all the patients, yet three patients remained with moderate to severe cognitive disability, and one patient, who was diagnosed with small cell lung cancer while admitted with status epilepticus and severe sepsis, passed away shortly after receiving the diagnosis. Only one patient had a clear relapse, with an excellent clinical response to renewed immunotherapy.

**GAD**

Four patients presented with encephalitis and showed high serum titers of anti GAD65 abs. Three were females (75%), median age at presentation was 34.5 years. Presenting symptoms included cognitive decline, encephalopathy, seizures (both GTCS and focal) and pain.

CSF showed normal cell counts and protein levels (n=3). 75% were positive for intrathecal synthesis of oligoclonal bands (n=3/4).

MRI was abnormal in two patients, showing mesiotemporal abnormalities. 75% were treated initially with steroids and a similar number with IVIG. Two patients required second line therapy, which consisted of Rituximab and Cyclophosphamide. All patients showed some degree of clinical improvement following immunomodulatory therapy, yet all remained on supportive treatment with anti-epileptic medication, benzodiazepines or baclofen. One patient that was diagnosed with progressive encephalopathy rigidity and myoclonus (PERM) died while awaiting stem-cell transplant, and one remained with significant disability. Interestingly, none had a preexisting diagnosis of diabetes, though one was admitted with severe DKA and diagnosed with diabetes upon admission.

**Unclassified**

Five patients had a positive immunofluorescence assay showing a distinct yet unrecognized pattern and fulfilled criteria for possible or definite AIE. Three patients were females, and the median age of onset was 54 years. Four patients presented with behavioral changes, of whom one showed signs of overt psychosis. Two patients had accompanying ataxia. The fifth patient presented with memory disturbances and episodes of unresponsiveness accompanied by gustatory symptoms suggestive of uncinate fits. Only one patient had abnormal MRI findings, showing bilateral mesiotemporal hyperintensities. All patients had elevated protein levels in their CSF (52-124 mg/dL), 80% had CSF pleocytosis (range 8-182 cells/µL), and 60% were positive for intrathecal synthesis of oligoclonal bands. None of the patients had a known malignancy, yet one was diagnosed with breast cancer following her AIE diagnosis. Response to therapy was less robust among this group with only 40% (2 patients) demonstrating clinical response to immune modulation.

**Seronegative (Table 4.)**

Twenty-two patients met criteria for definite, probable, or possible AIE, with no evidence of specific antibodies and a negative immunofluorescence assay. median age was 61 years (range 18-82), five were females (23%).

Presentation was acute in nine patients, subacute in eleven and chronic in two. The most common presenting symptoms were cognitive decline (45%), encephalopathy/confusion (32%) and behavioral changes (32%). Ataxia was reported in three patients. Epileptic manifestations, while uncommon as a presenting symptom (reported in four patients at presentation) were described throughout the acute illness in nine patients (41%).

EEG was overtly pathologic in twelve patients (54%) - four showed epileptiform activity and eight more had FIRDA, PLEDS, Triphasic waves and diffuse slowing.

Five patients had a known malignancy prior to diagnosis, and two were found to harbor a malignancy during follow-up (less than 1 year from diagnosis, one with testicular lymphoma and another one with AML).

Brain imaging was abnormal in 50% (11/22) of the patients, with the most common finding being unilateral or bilateral mesial temporal hyperintensities reported in five patients.
Lumbar puncture was pathologic in 82% (18/22). Pleocytosis was found in 59% (13/22), (range 6-330 cells/µL, median 0 cells/µL, mean 74 cells/µL), CSF protein was elevated in 73% (16/22) (range 21-208 mg/dL, median 80.5 mg/dL). Intrathecal synthesis of oligoclonal bands was positive in 23% (5/22), and a similar number had positive ANA. Of note, one patient was positive for anti SOX-1 in serum and CSF and later was found to have a cystic pulmonary lesion. This lesion was sampled with a nondiagnostic result.

Five patients (5/21) had serum leukocytosis. One had pneumonia, one had a normal blood count after 24 hours and normal infectious workup, one was due to concurrent steroid therapy upon admission and two presented shortly after a generalized seizure. Seven patients had hyponatremia (n=7/22, range 124-134 mmol/L, normal range 135.0-146.0 mmol/L). Six patients had mildly increased CRP (n=6/21, range 6-42 mg/L, normal range 0.03-5.00 mg/L), of them one had pneumonia, four had only minimal elevation ranging 6-9 mg/L, one had a high level of 42 mg/L and a normal infectious workup.

MoCA at presentation showed a median of 19.5 points (n=14, range 8-28, normal range 26-30). Patients tested both at admission and on follow-up showed a median improvement of 4.5 points with a median score at last follow-up of 24 (n=10).

All but one patient received immunomodulatory therapy. Following first-line therapy, eight patients required an additional second-line treatment 38.1% (n=8/21), of them five patients improved clinically, resulting in a total of seventeen patients who showed clear response (80%). Of the three remaining patients, one was stabilized, and two continued to deteriorate.

Seven patients (32%) had a follow-up mRS of 3-6, the rest (15 patients, 68%) had a follow-up mRS of 0-2 (Median MRS at follow-up was 1).

**ADEM**

After reviewing all our ADEM cases, we found seven cases manifesting as encephalitis. Four were females (57%), with a median age of 42 years (n=7, range 24-57). The most common presenting symptom was encephalopathy (6/7), followed by myelopathy (3/7), pyramidal signs and dysphagia (2/7 each). Two patients had optic neuropathy as well.

Brain imaging was pathologic in all our patients, most commonly showing diffuse white and gray matter Fluid-attenuated inversion recovery (FLAIR) hyperintensities.

Five patients had a pathologic lumbar puncture (n=6, 83%). All of them showed pleocytosis (mean 243 cells/µL, range 7-597 cells/µL, normal range 0-5 cells/µL), four of them also had an increased CSF protein (mean 94 mg/dL, range 54-211 mg/dL, normal range 12.0-60.0 mg/dL). Five patients were tested for anti-Aquapurin4 antibodies, all were negative. Only one patient was tested for MOG antibodies and was positive. CRP was mildly elevated in four patients (n=/46, range 8-27 mg/L, normal range 0.03-5.00 mg/L), serum leukocytosis was present in three patients (n=/36, range 15.3-20.5 10e3/µL, normal range 4.0-11.0 10e3/µL)

All patients were treated with first-line therapy during hospitalization (n=6/6), one patient required second line therapy for long term management. MoCA was not performed initially as patients were too impaired to cooperate. As a surrogate for functional assessment, we examined mRS. Median mRS at presentation was 4.5 points (range 1-5), while follow-up median mRS score was 2 (range 0-5).

**Hashimoto encephalopathy**

Five patients fulfilled criteria for Hashimoto encephalopathy, median age was 70 years (range 44-86), all were females. The most common presenting symptoms were encephalopathy, cognitive decline, seizures, myoclonus, and ataxia. All had a history of autoimmune thyroid disease.

Brain imaging was normal in all patients. One patient had a pathologic lumbar puncture with pleocytosis, and the three additional patients had increased protein levels (range 47-98 mg/dL) though elevation was extremely mild in two. We found no cases of intrathecal synthesis of oligoclonal bands. All patients had significantly increased anti-TPO antibodies, and three had also increased anti-thyroglobulin. There were no signs of systemic inflammation or electrolyte disturbances.

MoCA showed a median of 14 points (n=5, range 12-27) at presentation. Follow-up MoCA was available for two patients, and showed a median improvement of 4.5 points (n=2, range 20-25).

Most patients did well on first line therapy (60%, n=3/5). Two patients required a second line therapy.

**Additional specific antibodies**

Eight additional patients were found to carry specific AIE antibodies (GABA-B, Hu, Ri, PCA-2/MAP1B, caspr2, MA-2).

None had a proven paraneoplastic syndrome, though three were found to carry possible associated malignancies: The patient with GABA-B AIE had a mediastinal mass and passed away before malignancy workup was performed; an additional patient with Hu AIE presented with a suspicious paraesophageal lymph node on PET-CT, yet an endoscopic biopsy showed no evidence of malignant cells; and lastly, a patient with Ma-2 AIE was found to carry a testicular Leydig cell tumor, while Ma-2 is associated with germ-cell testicular tumors.

Response to immunomodulatory treatment was noted in six of the patients, yet the prognosis appears to be worse in this group, as 75% of patients had a follow-up mRS of 3 or higher, with two (25%) deceased.

Supplementary Tables

S1. Patients hospitalized in TLVMC per year.

| year | number of patients |
| --- | --- |
| 2010 | 77795 |
| 2011 | 77258 |
| 2012 | 77956 |
| 2013 | 74426 |
| 2014 | 76807 |
| 2015 | 77458 |
| 2016 | 77947 |
| 2017 | 76778 |
| 2018 | 79670 |
| 2019 | 83225 |
| 2020 | 74338 |

S2. Seronegative cases – clinical characteristics.

|  | **age** | **progression** | **clinical presentation** | **MRI** | **CSF** | **pathology** | **EEG** | **cancer** | **response to therapy** | **classification** |
| --- | --- | --- | --- | --- | --- | --- | --- | --- | --- | --- |
| 1 | 76 | acute | memory disturbances, dellusions, brief episodes of disconnection/absance | temporal lobe hyperintensity | normal | not done | epileptiform | no | did not receive immunotherapy | definite limbic encephalitis |
| 2 | 63 | subacute | cognitive decline, tremor, weight loss | widespread atrophy, mild temporal lobe hyperintensity | pleocytosis, elevated protein levels | not done | FIRDA | no | yes | probable seronegative |
| 3 | 42 | subacute | anxiety, brief episodes of disconnection/absance, GTCS | hyperintensity of limbic structures | OCB positive | not done | epileptiform | no | yes | definite limbic encephalitis |
| 4 | 18 | acute | seizures, encephalopathy | unremarkable | pleocytosis, elevated protein levels, OCB positive | not done | unremarkable, clinical generalized siezures | no | yes | possible AIE |
| 5 | 64 | subacute | confusion, tremor | diffuse white matter changes with restricted diffusion of basal ganglia | pleocytosis, elevated protein levels, OCB positive | uninformative | unremarkable | no | yes | probable seronegative |
| 6 | 59 | subacute | confusion, GTCS | microangipatic changes | elevated protein levels | not done | epileptiform | no | yes | possible AIE |
| 7 | 70 | acute | recurrent focal deficits, suspected clinical focal siezures | unremarkable | mild pleocytosis, elevated protein levels | not done | unremarkable, suspected clinical focal siezures | no | yes | possible AIE |
| 8 | 32 | subacute | 2 months of headaches, sensory disturbances, aphasia, followed by acute severe confusion and aggitation | unremarkable | pleocytosis, elevated protein levels | not done | unremarkable (under general anasthesia) | no | yes | possible AIE |
| 9 | 35 | acute | severe headaches and confusion | unremarkable | pleocytosis, elevated protein levels | not done | bilateral frontotemporal slowing, FIRDA, clinical GTCS | no | yes | possible AIE |
| 10 | 36 | acute | confusion, myoclonus, GTCS | unremarkable | pleocytosis, elevated protein levels | not done | moderate left frontotemporal slowing, clinical siezures | no | yes | possible AIE |
| 11 | 64 | subacute | gait disturbances followed by rapidly progressive cognitive decline, acute short episode of aphasia | microangipatic changes, cortical and subcortical ischemic lesions | minimal pleocytosis, elevated protein levels | not done | generalized bilateral slowing, prominent Delta on left fronto-temporal | no | yes | possible AIE |
| 12 | 82 | chronic | cognitive difficutlies and and gait disturbances, evidence of CIDP | atrophy, suspected NPH | elevated protein levels | not done | not done | mds-cmml, highly suspicious mass but two biopies uninformative | yes | possible paraneoplastic |
| 13 | 78 | chronic | cognitive decline | microangipatic changes, cortical and subcortical ischemic lesions | elevated protein levels | not done | unremarkable | diagnosed shortly after with DLBCL | no improvement, progression stopped | possible paraneoplastic |
| 14 | 71 | subacute | opsoclonus, confusion, ataxia, fever and diffuse body aches | unremarkable | normal (OCB was not done) | not done | not done | no | yes | possible AIE |
| 15 | 45 | subacute | confusion, seizures | hydrocephalus followed by diffuse white matter changes | pleocytosis, OCB positive, Flow cytometry show no evidence of monoclonality | microglial nodular encephalitis | unremarable | follicular lymphoma | no | possible paraneoplastic |
| 16 | 47 | subacute | edisodic headaches with sensory disturbances and aphasia, then acute confision, hemiparesis, generalized seizure | few hyperintense subcortical small lesions | pleocytosis, elevated protein levels | not done | not done | no | yes | possible AIE |
| 17 | 56 | acute | confusion | hyperintensity and swelling of brainstem including both thalami and right prietal cortical region | pleocytosis, elevated protein levels | not done | mild to moderate diffuse slowing | 2 yrs after the acute eposide diagnosed with AML | yes | definite paraneoplatic |
| 18 | 35 | acute | dysarthria and ataxia - cerebellar syndrome | mild hyperintensity and swelling of the cerebellum | pleocytosis, OCB positive | not done | not done | no | no | possible AIE |
| 19 | 40 | acute | confusion | a hyperintensem restrictive, ring enhancing lesion in the corpus collosum (suspected post ictal) | mild pleocytosis | not done | diffuse delta and theta slowing | no | yes | possible AIE |
| 20 | 72 | subacute | memory disturbances, cognitive decline | bilateral mesiotemporal hyperintensities | normal. OCB negative | not done | FIRDA | metastatic carcinoid | yes | possible AIE |
| 21 | 71 | acute | confusion, dellusions, psychosis following treatment with anti-PD1 | microangiopathic changes | elevated protein levels, | not done | mild left temporal slowing | metastatic RCC treated with anti-PD1 | yes | possible AIE |
| 22 | 67 | subacute | memory problems | hypocampal asymmetry | normal, OCB negative | not done | left temporal epileptiform activity | no | yes | possible AIE |

Table S2 Legend:

AIE – autoimmune encephalitis, AML – acute myelocytic leukemia, CIDP - Chronic inflammatory demyelinating polyneuropathy, DLBCL – diffuse large B cell lymphoma, GTCS – generalized tonic-clonic seizure, FIRDA – frontal intermittent rhythmic delta activity, MDS-CMML - myelodysplastic syndrome chronic myelomonocytic leukemia, OCB – oligoclonal bands, PD1 - Programmed cell death protein-1.

S3. Infectious encephalitis breakdown of causes:

| **Viral** | **total 95** |
| --- | --- |
| EBV | 2 |
| Enterovirus | 1 |
| HHV-6 | 3 |
| HIV | 2 |
| HSV-1 | 16 |
| HSV-2 | 5 |
| TBEV | 1 |
| Influenza | 2 |
| VZV | 30 |
| WNV | 33 |
| **Bacterial** | **total 41** |
| Listeria monocytogenes | 12 |
| Neisseria meningitidis | 1 |
| Streptococcus pneumoniae | 23 |
| Rickettsia | 1 |
| Q-Fever | 1 |
| Streptococcus pyogenes | 1 |
| E.Coli | 1 |
| Klebsiella pneumoniae | 1 |
| **Fungal** | **total 10** |
| Toxoplasmosis | 7 |
| Cryptococcus | 2 |
| Aspergillus | 1 |
